# Concentrations of trace elements in human milk: Comparisons among women in Argentina, Namibia, Poland, and the United States

**DOI:** 10.1371/journal.pone.0183367

**Published:** 2017-08-17

**Authors:** Laura D. Klein, Alicia A. Breakey, Brooke Scelza, Claudia Valeggia, Grazyna Jasienska, Katie Hinde

**Affiliations:** 1 Department of Human Evolutionary Biology, Harvard University, Cambridge, Massachusetts, United States of America; 2 Department of Anthropology, University of California Los Angeles, Los Angeles, California, United States of America; 3 Department of Anthropology, Yale University, New Haven, Connecticut, United States of America; 4 Department of Environmental Health, Faculty of Health Sciences, Jagiellonian University, Krakow, Poland; 5 Center for Evolution and Medicine, Arizona State University, Tempe, Arizona, United States of America; 6 School of Human Evolution and Social Change, Arizona State University, Tempe, Arizona, United States of America; University of PECS Medical School, HUNGARY

## Abstract

Human milk contains essential micronutrients for growth and development during early life. Environmental pollutants, such as potentially toxic metals, can also be transferred to the infant through human milk. These elements have been well-studied, but changing diets and environments and advances in laboratory technology require re-examining these elements in a variety of settings. The aim of this study was to characterize the concentrations of essential and toxic metals in human milk from four diverse populations. Human milk samples (n = 70) were collected in Argentina (n = 21), Namibia (n = 6), Poland (n = 23), and the United States (n = 20) using a standardized mid-feed collection procedure. Milk concentrations of calcium, zinc, iron, copper, manganese, lead, arsenic, and cadmium were determined using inductively coupled plasma mass spectrometry (ICP-MS). We used standard multiple linear regression models to evaluate differences among populations, while including infant age, infant sex, and maternal parity status (multiparous or primiparous) as covariates. Concentrations of all elements, except zinc, varied across populations after controlling for infant age, infant sex, and maternal parity. Calcium and magnesium showed more differences across populations than iron or copper. There were no significant differences among population in zinc concentrations. Mean concentrations of lead, but not arsenic, were low compared to recently published values from other populations. The concentrations of trace elements in human milk are variable among populations. Limitations due to small sample sizes and environmental contamination of some samples prevent us from drawing robust conclusions about the causes of these differences.

## Introduction

Human milk is considered the ideal food for infant nutrition. In addition to macronutrients, human milk also contains micronutrients, including trace minerals. Many of these elements are essential for growth and development during early life as micronutrient deficiencies during early life adversely affect individual and community health [1]. Micronutrient deficiencies are associated with more frequent infections in the short-term and increased rates of chronic diseases and reduced work productivity in the long-term [2]. However, excessive amounts of these elements can also be detrimental. High levels of iron in formula may increase an infant’s risk of infection by increasing nutrient bioavailability to pathogenic bacteria [3], and high manganese exposure in children has been associated with impaired cognitive development and motor coordination [4]. Determining appropriate intake levels of micronutrients during early life is of substantial importance for public health recommendations and clinical interventions. Thus, exploring the variation of milk bioactives in human milk within and across populations is particularly necessary [5].

In addition to essential elements, milk can also transfer potentially toxic metals, such as lead, arsenic, and cadmium. These metals have been detected in milk around the world, though concentrations can vary widely depending on environmental exposures like diet, the use of leaded gasoline, or smoking [6]. Toxic metals do not generally accumulate in human milk even when present in maternal circulation and postnatal exposure via milk ingestion is likely lower than fetal exposure [7,8]. Early-life exposures, however, can contribute to long-lasting adverse health outcomes, such as neurodevelopmental disorders [8] and impaired immune and respiratory function [9]. Moreover, environmental exposures to toxic metals often disproportionately affect marginalized groups [10, 11]. Concerns about pollutants in human milk can also affect infant feeding decisions [10].

Although decades of research effort has been allocated to human milk concentrations of micronutrients and toxic metals, dietary shifts, environmental changes, and more sensitive analytical technologies motivate sustained investigation [12]. For example, human milk lead levels in Sweden decreased significantly from 1989 to 2009, likely as a result of the ban on leaded gasoline use [12]. However, levels of toxic elements in human milk remain high in many areas around the world [13, 14]. Cinar and colleagues [13] reported that some of the highest levels of toxic metals in human milk in Turkey were found in rural, not urban or industrial areas. In the US and Canada, tribal lands are protected by fewer environmental regulations than non-tribal lands, and experience greater environmental degradation and pollution [10]. Many traditional populations are experiencing varying degrees of market integration or urbanization, which can be associated with changes in diet and/or environmental exposures [15–18].

Due to the importance of adequate micronutrient intake in early life for individual and community health, and changing diets and environments worldwide, the study of human milk trace elements remains relevant to research in human lactation and public health. Here we characterize the concentrations of five essential and three toxic elements in human milk as it is typically consumed across diverse populations from Argentina, Poland, the US, and Namibia. Samples from Argentina and Namibia were collected in indigenous populations. Previous studies have understandably focused on populations that are severely malnourished, at high risk for toxic metal exposure, or are in urban areas of wealthy nations. By contrast, the range of geography and lifestyle represented by the populations in this study attempts to reflect the breadth of modern human environments, including populations that are not well-represented in the current literature.

## Methods

Samples for this project were collected as part of a larger investigation of the composition of human milk across diverse populations. A standardized collection procedure was used to facilitate comparison across populations. Participants for whom at least 2 ml of milk sample remained after other planned analyses were included in present report. Milk samples were divided into aliquots shortly after collection, thus, the composition of the subsample analyzed here is expected to be an unbiased representation of the full sample from each individual. Study procedures were approved by the Harvard Committee on the Use of Human Subjects (#23868, #21979, #13–0900), the University of Pennsylvania Institutional Review Board (#811200), and the University of California Los Angeles Institutional Review Board (#13–000881). No formal ethical approval was required at the local level in Argentina or Poland, though permission to conduct research was obtained from the village priest in Poland. Research in Namibia was conducted under research visa (#W830312013) and local approval was also granted by the Chief of the area. Written informed consent was obtained from all participants except those in Namibia, where informed oral consent was obtained from each participant and the participant’s head of household because the population is illiterate. Documentation of oral consent was not required, as it was implicit that the start of the oral interview meant that oral consent had been given.

### Participants

Milk samples were provided by lactating mothers (N = 70) (Table 1). Inclusion criteria were mothers nursing biological offspring produced from a singleton pregnancy and no indication of mastitis at the time of milk collection. Mothers of infants under two weeks of age or over two years of age were excluded from the study. No participants were current smokers.

**Table 1:** Demographic characteristics of sample.

	United States	Namibia	Poland	Argentina	Total
	(n = 20)	(n = 6)	(n = 23)	(n = 21)	(N = 70)
Infant Sex					
Male	7	2	12	9	30
Female	13	4	11	12	40
Infant Age, days *M*(*SD*)	200 (101)	239 (194)	195 (100)	241 (107)	214 (112)
Maternal Parity Status					
Primiparous	10	0	11	3	24
Multiparous	10	6	12	18	46
Parity *M*(*SD*)	1.7 (0.9)	5 (3.1)	2.1 (1.2)	3.9 (2.6)	2.8 (2.1)

### Settings

Samples were collected in a variety of contexts. Women living in the Boston area provided human milk samples from June to August 2013 and represent an urban W.E.I.R.D. (Westernized, educated, industrial, rich, democratic) population [19]. Polish samples were collected at the Mogielica Human Ecology Study Site, a group of rural villages in southern Poland [20], during July and August 2012. This region has historically engaged in small-scale agriculture, but is increasingly transitioning to participation in wage labor. Most people live in modern houses and all have access to professional health care [21, 22]. Argentinean samples were collected from indigenous Qom (formerly Toba) women in northeastern Argentina from September 2012 to March 2013. Traditionally, the Qom people were hunter-gathers, but today many have migrated to poor peri-urban barrios where they have access to free governmental healthcare, but often share outdoor water taps and lack indoor toilets [23, 24]. Namibian samples were collected from indigenous Himba women living in northern Namibia during September 2013. The Himba people are semi-nomadic agro-pastoralists. There is one small medical clinic a day’s walk from the study area and a communal water tap, but the community lacks plumbing and electricity [25, 26].

### Data collection

#### Demographic information

Maternal parity, infant age, and infant sex were self-reported by the mother during guided oral interviews in the participant’s native language.

#### Milk collection

A single, mid-feed milk sample was provided by each mother [27]. Samples were collected between 8AM and 11:30AM. Participants were asked not to feed the infant from the sample breast for approximately 2 hours before collection. Participants self-expressed by hand a milk sample up to 10 ml into polypropylene BD Falcon tubes (#352070). Samples were then gently mixed by hand and aliquoted into Axygen cryovials (#22–269). Samples were frozen at -20°C (in Argentina and Namibia) or -80°C (in Poland and the US) shortly after collection. Samples from outside the US were shipped to Harvard University on dry ice and frozen at -80°C until analysis. Mineral and metal content is stable through freeze/thaw cycles [28].

### Data analysis

#### Milk trace metal analysis

Milk calcium (Ca), zinc (Zn), iron (Fe), copper (Cu), manganese (Mn), arsenic (As), lead (Pb), and cadmium (Cd) in milk were analyzed at The Trace Metals Lab at the Harvard School of Public Health. Briefly, 1 to 2 ml of sample was measured on an analytical balance and 1 mL of ultrapure nitric acid (BDH Aristar Ultra) was added to each sample. Samples were then digested with a Milestone Ultrawave microwave digestion system, diluted with deionized water to a final volume of 10 ml, and analyzed with a Perkin Elmer ELAN DRC II ICP Mass Spectrometer. Cadmium levels were below the level of detection for all samples and therefore excluded from analysis.

#### Statistical analysis

Population differences in metal levels were evaluated by standard multiple linear regression models. All analyses were conducted in R version 3.3.0. Pearson correlation coefficients and p-values were determined using the “Hmisc” package. Data were graphically inspected for normality. To normalize data distributions, Fe, Pb, and As concentrations were Box-Cox transformed and Zn, Cu, and Mn concentrations were natural log transformed before analysis. Calcium concentrations were approximately normally distributed and thus not transformed. Infant age (in days), infant sex, and maternal parity (coded as multiparous or primiparous) were included as covariates in all models. Post-hoc pairwise comparisons were conducted with the “lsmeans” package and adjusted for multiple comparisons using the Holm method. Alpha was set at 0.05 and all p-values presented are two-tailed.

## Results

### Correlations among trace elements

To determine whether differences in milk micronutrient levels between populations might be due to a dilution effect, correlations among the trace elements were calculated. Correlations between trace element concentrations across all 70 samples ranged from weakly negative to moderately positive. Calcium was positively correlated to iron (*r* = 0.41, *p*<0.001) and copper (*r* = 0.42, *p*<0.001) but negatively correlated with lead (*r* = -0.36, *p*<0.001) and arsenic (*r* = -0.3, *p* = 0.01). Zinc was positively correlated to copper (*r* = 0.39, *p* = 0.001). Lead was positively correlated with iron (*r* = 0.29, *p* = 0.016), manganese (*r* = 0.26, *p* = 0.03), and arsenic (*r* = 0.29, *p* = 0.01). Arsenic was also positively correlated to manganese (*r* = 0.4, *p*<0.001).

### Infant and maternal characteristics

Calcium, iron, zinc, and copper concentrations in milk decreased with infant age (Ca: *β* = -0.46, *t* = -5.39, *p*<0.0001; Fe: *β* = -0.50, *t* = -5.36, *p*<0.0001; Zn: *β* = -0.57, *t* = -5.63, *p*<0.0001; Cu: *β* = -0.58, *t* = -6.20, *p*<0.0001). Infant age was not significantly associated with manganese, lead, or arsenic concentrations. Infant sex did not significantly predict mineral concentrations in any model (*p*≥0.10 for all). Similarly maternal primiparity was not associated with trace mineral concentration in milk, although primiparous mothers tended to produce milk with higher levels of iron than did multiparous mothers (*M* ± *SD*: 1.17 ± 0.28 mg/L vs 1.10 ± 0.38 mg/L, respectively, *t*(60) = 1.89, *p* = 0.06).

### Differences among populations

The concentrations of elements in human milk were variable, both within and among populations (Table 2). All comparisons are controlled for infant age, sex, and maternal parity status (primiparous or multiparous). The trace mineral profile of human milk samples often differed among populations, but not always in the same directions (Table 3).

**Table 2:** Summary of trace element concentrations.

	United States	Namibia	Poland	Argentina	Total
Trace Element	(n = 20)	(n = 6)	(n = 23)	(n = 21)	(N = 70)
Calcium (mg/L)					
*M*	268.72	143.83	227.06	231.79	233.25
*SD*	59.34	64.67	36.72	37.62	56.45
Min	138.02	36.69	152.94	177.47	36.69
Max	374.95	205.21	293.71	304.47	374.95
Iron (mg/L)					
*M*	1.27	1.53	1	0.99	1.12
*SD*	0.26	0.86	0.15	0.21	0.35
Min	0.84	0.74	0.8	0.71	0.71
Max	1.85	2.97	1.38	1.51	1.38
Zinc (mg/L)					
*M*	0.67	1.34	0.75	0.93	0.83
*SD*	0.43	1.29	0.46	0.5	0.59
Min	0.15	0.03	0.2	0.25	0.03
Max	1.61	3.75	2.02	2.01	3.75
Copper (μg/L)					
*M*	169.52	130.94	186.87	211.04	184.4
*SD*	63.06	63.49	48.1	99.5	74.33
Min	71.48	55.6	82.95	89.52	55.6
Max	317.09	208.83	252.42	419.09	419.09
Manganese (μg/L)					
*M*	2.71	11.6	1.61	7.62	4.58
*SD*	1.12	9.78	0.89	3.76	4.76
Min	1.46	2.79	0.22	3.29	0.22
Max	5.86	30.27	4.32	20.24	30.27
Arsenic (μg/L)					
*M*	3.47	6.68	3.86	4.51	4.18
*SD*	0.84	2.46	1	1.34	1.5
Min	2.4	4.08	3.03	2.54	2.4
Max	6.02	11.2	7.9	9.08	11.2
Lead (μg/L)					
*M*	0.77	2.15	1.02	0.59	0.91
*SD*	0.45	0.24	0.26	0.4	0.55
Min	0.41	1.92	0.52	0.21	0.21
Max	2.1	2.48	1.44	1.69	2.48

**Table 3:** Pairwise comparisons of trace element concentrations between populations.

	Calcium		Zinc		Iron		Manganese		Copper		Arsenic		Lead	
Contrast	*t* (63)	*p*	*t* (63)	*p*	*t* (63)	*p*	*t* (63)	*p*	*t* (63)	*p*	*t* (63)	*p*	*t* (63)	*p*
US—Namibia	5.62	< .001	-1.43	0.634	-0.99	0.653	-5.27	< .001	1.02	0.627	-5.28	< .001	-3.01	0.013
US—Poland	3.53	0.003	-0.51	1.000	4.01	0.001	3.74	< .001	-0.96	0.627	-1.83	0.072	-2.20	0.062
US—Argentina	1.70	0.187	-2.60	0.070	2.81	0.020	-6.16	< .001	-2.83	0.032	-3.81	0.001	3.06	0.013
Namibia—Poland	-3.43	0.003	1.12	0.803	3.57	0.003	7.75	< .001	-1.65	0.314	4.19	< .001	1.64	0.107
Namibia—Argentina	-4.68	< .001	-0.36	1.000	3.05	0.014	1.13	0.261	-3.09	0.018	2.82	0.019	5.34	< .001
Poland—Argentina	-1.63	0.187	-2.20	0.156	-0.95	0.653	-9.98	< .001	-2.00	0.198	-2.19	0.065	5.29	< .001

**Fig 1:**
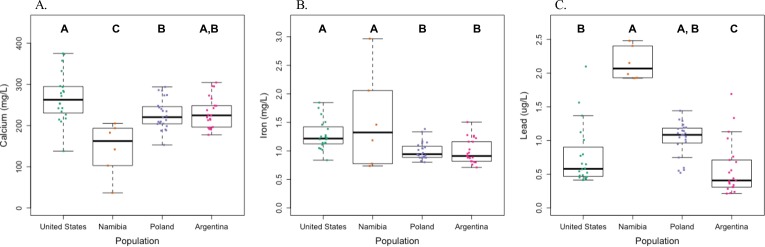
Calcium, iron, and lead in breast milk from four populations. This figure illustrates the concentrations of (A) calcium, (B) iron, and (C) lead measured in breast milk in samples from the US, Namibia, Poland, and Argentina. Each point illustrates an individual sample value, and the bold horizontal line in each box plot depicts the median for each population. Letters indicate statistically significant differences among population means (p< 0.05).

Of the essential trace elements, calcium and manganese differed most among populations. Calcium concentrations were significantly lower in the Namibian population than all others (US: *p*<0.0001; Poland: *p* = 0.003; Argentina: *p* = 0.001) (Fig 1). US milk samples had higher calcium levels than did samples from Poland (*p* = 0.003), but not Argentina. Mean manganese concentrations were 3 to 4 times higher in the Namibian and Argentinian samples compared to the US (US-Namibia: *p* = < .0001; US-Argentina: *p* = 0.0008) or Polish (Poland-Namibia: *p* = <0.0001; Poland-Argentina: *p* = <0.0001) samples. There was no significant difference in manganese concentrations between the Namibian and Argentinian samples, but the US had higher levels than the Polish (*p* = 0.0008) samples.

Iron, zinc, and copper showed fewer differences among populations. Iron levels in the US and Namibian samples were higher than those from Poland (US-Poland: *p* = 0.001; Namibia-Poland: *p* = 0.003) and Argentina (US-Argentina: *p* = 0.019; Namibia-Argentina: *p* = 0.01) (Fig 1). However, iron levels were not significantly different between US and Namibian samples or Polish and Argentinian samples. There were no significant differences in zinc concentration among populations, though concentrations in the Argentinian samples tended to be higher than in the US samples (*p* = 0.07). Copper concentrations were highest in the Argentinian samples, and were significantly higher than in the US (*p* = 0.03) and Namibian (*p* = 0.018), but not Polish (*p* = 0.19) samples. There were no significant differences in copper concentrations among the US, Polish, and Namibian samples (all *p*≥0.19).

Arsenic and lead were present in samples from all populations. Namibia had significantly higher arsenic concentrations than Argentina (*p* = 0.0191), Poland (*p* = <0.001) and the US (*p* = <0.0001). US arsenic concentrations were significantly lower than Argentinian (*p* = 0.001) samples, but were not significantly different from the Polish (*p* = 0.07). There was also no significant difference between the Polish and Argentinian (*p* = 0.06) samples. Lead levels were significantly lower in the Argentinian samples compared to all other populations (US: *p* = 0.01; Poland: *p*<0.0001; Namibia: *p*<0.0001) (Fig 1). The US had lower lead levels than Namibia (*p* = 0.01), but there was no significant difference between lead concentrations in Poland and the US (*p* = 0.06) or Namibia (p = 0.11).

## Discussion

In this study we characterized the concentrations of five trace elements (Ca, Zn, Fe, Cu, Mn) and two toxic elements (As, Pb) across diverse populations in the United States, Argentina, Poland, and Namibia. Calcium, iron, zinc, and copper concentrations decreased across lactation, but there was no relationship between infant age and manganese, lead, or arsenic. Neither infant sex nor maternal parity status was significantly associated with any trace mineral concentration. The concentrations of elements in human milk were variable both within and among populations, and no population had consistently higher or lower levels of the essential or toxic elements. Calcium and manganese differed more among populations while zinc, iron, and copper showed no or fewer differences among populations, perhaps reflecting common physiological mechanisms to avoid severe deficits of these essential micronutrients. Arsenic and lead were present in samples from all populations.

Calcium and manganese concentrations differed more among populations compared to other essential trace elements in the present study. This is consistent with previous reports that have found high variation in the reported values of both elements (Table 4). Previously reported mean calcium concentrations have ranged from 84 to 462 mg/L, with a median of 252 mg/L [29]. Population-level mean calcium concentrations (Table 2) in this study fell within this range and were similar to unsupplemented Gambian mothers [30] and slightly less than recently reported values from Swedish mothers [12]. Lower values observed here could be explained in part by the older ages of infant included in the present study, as calcium concentrations in milk progressively decrease after 12 weeks post-partum [31]. Samples in this study were collected between 2 weeks and 17 months postpartum, while the Swedish and Gambian samples were collected at 2–3 and 13 weeks postpartum, respectively. In contrast to calcium, manganese concentrations in milk are very low (4–8 μg/L) [32]. Ranges in manganese concentrations in this study showed 3 to 4 fold differences among populations, which is consistent with previous studies [33]. Notably, manganese was the only essential trace element in our study that was not significantly related to infant age. Casey and colleagues [34] reported that manganese concentrations became irregular during gradual weaning. Since the average age of all infants in our study is approximately 7 months (*M* ± *SD*: 214 ± 111 days), most are expected to be receiving complementary foods and are likely to be at various stages of the weaning process.

**Table 4:** Trace element concentrations reported in mature human milk.

Trace Element	Country	Analysis Method^a^	n	Median	*M* ^b^	*SD*	Unit	Reference
Calcium								
	Brazil	ICP-AES	31		250	31	mg/L	[35]
	Gambia	Methyl thymol blue	120		208.93^c^	24	mg/L	[29]
	Japan	ICP-AES	1170		250	71	mg/L	[36]
	Nigeria	Thermo Labsystems Arsenazo III Kit	105		186	41	mg/L	[37]
	Sweden	ICP-MS	60		305	45	mg/L	[12]
	US	AAS	20		279.2	127.90	mg/L	[38]
Copper								
	Brazil	AAS	116		0.3	0.1	mg/L	[39]
	Honduras	AAS	105		0.16	0.21	mg/L	[40]
	Japan	ICP-AES	1169		0.35	0.21	mg/L	[36]
	Kuwait	AAS	17		0.608^c^	0.027	mg/L	[41]
	Poland	GF AAS	320		0.137	0.092	mg/L	[42]
	Sweden	AAS	86		0.12	0.22	mg/L	[40]
	Sweden	ICP-MS	60		0.471	0.075	mg/L	[12]
	Turkey	ICP-OES	75		0.446^c^	0.197	mg/L	[13]
	US	AAS	30		0.27	0.11	mg/L	[38]
	Vietnam	ICP-AES	59		0.19	0.05	mg/L	[43]
Iron								
	India	AAS	16		0.168^c^	0.406	mg/L	[44]
	Brazil	ICP-AES	31		0.9	0.5	mg/L	[35]
	Brazil	AAS	116		0.3	0.2	mg/L	[39]
	Honduras	AAS	105		0.21	0.25	mg/L	[40]
	Japan	ICP-AES	1155		1.19	2.51	mg/L	[36]
	Japan	AAS	24		0.32	0.16	mg/L	[45]
	Kuwait	AAS	17		0.4^c^	0.040	mg/L	[41]
	Sweden	AAS	86		0.29	0.21	mg/L	[40]
	Sweden	ICP-MS	60		0.339	0.134	mg/L	[12]
	Turkey	ICP-OES	75		1.072^c^	0.841	mg/L	[13]
	US	AAS	41		0.36	0.19	mg/L	[38]
	Vietnam	ICP-AES	59		0.43	0.15	mg/L	[43]
Manganese								
	Brazil	ICP-MS	58	0.33			ug/L	[46]
	Japan	ICP-AES	1167		11	23	ug/L	[36]
	Japan	AAS	24		9.5	6.3	ug/L	[45]
	Kuwait	AAS	17		4.71^c^	0.16	ug/L	[41]
	Sweden	ICP-MS	60		3	11.4	ug/L	[12]
	Turkey	ICP-OES	74		124^c^	156	ug/L	[13]
	US	AAS	116		4.9	3.9	ug/L	[33]
Zinc								
	Brazil	ICP-AES	31		1.5	0.6	mg/L	[35]
	Brazil	AAS	116		2.7	1.3	mg/L	[39]
	Brazil	ICP-MS	58	0.0462			mg/L	[46]
	Honduras	AAS	105		0.7	0.18	mg/L	[40]
	India	AAS	50	2.5			mg/L	[47]
	India	AAS	47	1.37			mg/L	[47]
	India	AAS	50	1.17			mg/L	[47]
	Japan	ICP-AES	1165		1.45	1.35	mg/L	[36]
	Kuwait	AAS	17		2.56^c^	0.136	mg/L	[41]
	Poland	GF AAS	320		1.62	1.76	mg/L	[42]
	Sweden	AAS	86		0.46	0.26	mg/L	[40]
	Sweden	ICP-MS	60		3.471	0.979	mg/L	[12]
	Turkey	ICP-OES	75		3.454	1.970	mg/L	[13]
	US	AAS	30		1.45	1.37	mg/L	[38]
	Vietnam	ICP-AES	59		0.56		mg/L	[43]
Arsenic								
	Croatia	ICP-MS	123	0.2			ug/L	[48]
	Greece	ICP-MS	39	0.8			ug/L	[48]
	Italy	ICP-MS	602	0.3			ug/L	[48]
	Japan	ICP-MS	9	1.4			ug/L	[49]
	Slovenia	ICP-MS	287	0.4			ug/L	[48]
	Sweden	ICP-MS	60		0.33	0.041	ug/L	[12]
	Taiwan	AAS	90		0.215^c^	0.81	ug/L	[50]
Lead								
	Brazil	ICP-MS	58	0.26			ug/L	[45]
	Iran	AAS	37		7.11	3.96	ug/L	[51]
	Iraq	AAS	32		31.65	22.19	ug/L	[14]
	Iraq	AAS	36		19.59	13.66	ug/L	[14]
	Japan	ICP-MS	9	0.29			ug/L	[49]
	Palestine	GF AAS	89	4			ug/L	[11]
	Poland	GF AAS	320		6.33	4.61	ug/L	[42]
	Sweden	ICP-MS	60		1.5	0.9	ug/L	[12]
	Taiwan	AAS	90		17.17^c^	2.18	ug/L	[50]
	Turkey	ICP-OES	56		261^c^	171	ug/L	[13]

^a^ Abbreviations: AAS = Atomic Absorption Spectroscopy, GF AAS = Graphite Furnace Atomic Absorption Spectroscopy, ICP-AES = Inductively Coupled Plasma Atomic Emission Spectroscopy, ICP-MS = Inductively Coupled Plasma Mass Spectrometry, ICP-OES = Inductively Coupled Plasma Optical Emission Spectrometry

^b^ Means have been standardized to a common unit for each element.

^c^ Weighted population mean calculated with data reported for sub-groups.

Iron, zinc, and copper tended to be more consistent among populations. Unlike calcium, which is largely associated with citrate or casein in human milk [30], these metals tend to be bound to milk proteins. Iron is largely bound to fat-globule associated proteins or lactoferrin [37, 52], while zinc and copper tend to be bound to whey proteins, including serum albumin [37]. Human milk is typically low in iron, with a mean around 0.6 mg/L in early lactation that steadily decreases to a mean between 0.2–0.3 mg/L after 5–6 months [53]. In the present study, however, mean population concentrations were 1.5 to 4 fold higher. Values in our study are higher than most previously reported values, but are similar to values from Turkey [13] but are still 1.5 to 7 fold lower than typical infant formula levels in Europe or the US [53]. We found that primiparous mothers produced slightly higher concentrations of milk iron than did multiparous mothers. This is consistent with previous studies report no relationship between parity and iron concentrations in mature milk [54, 55]. Our results are generally consistent with studies that reported no differences based on location, race, or population or attributable to differences in milk volume ([40, 56] but see [57, 58]). Milk copper levels in this study fell within the range of previously reported concentrations from Sweden [40], Honduras [40], the US [38], and Brazil [39]. The consistency of concentrations across populations despite the range of geography and lifestyle likely reflects the importance of these trace elements for proper development and function, and common physiological mechanisms to maintain adequate levels for the infant.

Mean population levels of toxic metals were low across all populations in this study. The World Health Organization set a safety limit of human milk lead concentration between 2 and 5 μg/L [59]. In this study, only Namibia had a mean lead concentration greater than 2 μg/L. Lead concentrations are comparable to mean levels found in mature milk in Sweden [12] and industrial areas in Taiwan [50]. Lead concentrations in this study are also lower than recently reported values from urban and rural areas in Iraq [14] and Turkey [13]. Mean arsenic concentrations were all below the EPA and WHO recommended limit of 0.01 mg/L in drinking water [60]. However, the values we observed are 2 to 5 fold higher than recently published values from Taiwan and Sweden, which both found milk arsenic concentrations <1 μg/L [50, 12].

Population differences in the concentrations of these trace elements, particularly the toxic metals, may be due to differences in environmental exposures. Arsenic exposure primarily occurs through drinking water or food, and naturally high groundwater levels of arsenic occur in several countries, including Argentina and the US [61]. Higher levels of lead in human milk have been reported in women that live closer to industrial [51, 50] or urban areas [62], or use cosmetics containing lead [11]. However, risk factors for toxic metal exposure were not directly assessed in this study. Dietary differences among populations are unlikely to explain much of the variation observed in this study. Milk manganese concentrations covaried with maternal dietary intake in one study [63] but iron, zinc, and copper have not been found to relate to maternal dietary intake [1, 32]. Similarly, most studies have not found an effect of dietary intake on milk calcium levels [32].

Population differences in essential milk element concentrations may represent local adaptations to their immediate environments [64]. Understanding local adaptations has direct implications for the clinical management of public health interventions. More than 2 billion people worldwide are estimated to have micronutrient deficiencies [65], and supplementation efforts to improve health outcomes, particularly around pregnancy and lactation, are a major focus of public health research [65–67]. However, appropriate levels of supplementation may not be uniform across populations and supplementation interventions have had unintended adverse consequences. For example, Gambian women had lower bone density during and for years after lactation after receiving calcium supplementation during pregnancy [30]. Moreover, there were no discernable benefits for infant health [30]. In another study, Kenyan infants who received iron-fortified porridge experienced higher levels of intestinal pathogens and inflammation [68]. Public health interventions are motivated by the best intentions to improve the current health of individuals and communities, but must consider local adaptations and intergenerational effects to be successful in the long-term.

This report represents several limitations of scope. As part of a larger research effort measuring multiple milk bioactives, only a subset of participants provided sufficient volume for all planned analyses, biasing inclusion toward participants producing the highest milk volume at the time of sample collection. This assessment of volume, however, remains relative and not absolute, as challenging research settings complicate reliable, standardized measurement of milk volume. Mid-feed milk samples, rather than full mammary evacuations, were used to minimize the nutritional impact to the infant in potentially nutritionally-stressed populations in our study [27]. Reliable volume measures would better enable us to speak to total potential transfer to the infant, which may be a more biologically meaningful measure, as milk volume may be more sensitive to changes in maternal condition than milk composition [69, 70]. However, if population differences in trace metal concentrations reflected a dilution effect, we would expect concentrations of all elements to be positively correlated. Correlation coefficients of mineral concentrations in our study, however, ranged from negative to positive. This suggests our results are unlikely to be explained as simply a dilution effect, though more systematic analysis using a marker protein concentration would be required to definitively eliminate this possibility. These correlations are also unlikely to be a byproduct of differences in casein content. Casein micelles in milk contain the majority of milk’s calcium content, but less than 15% of the other essential minerals [1]. Moreover, comparisons of mineral concentrations across populations did not reveal consistent patterns of higher or lower concentrations, which suggests that these differences are not due to different mean volumes across populations.

The Namibian samples included in this study require special considerations. Concentrations of most elements measured in this study, with the exceptions of calcium and copper, were highest in the Namibian samples. The sample size for this population, however, was small (n = 6) and some Namibian milk samples were visibly “contaminated” with *otjize*. A traditional cosmetic paste made of clarified butter (or when that is unavailable, petroleum jelly) and red ochre, *otjize* is applied daily to the hair and skin, including the breasts [71]. Red ochre gets its coloration from iron oxides, though the composition of ochres differs depending on its geographic origin [72]. Ochre used by the Himba is mainly composed of iron-ore, with trace amounts of other elements, including calcium, manganese, and copper [73, 74]. Ochres have been utilized extensively across cultures and throughout human history for both symbolic and practical uses, including by the Himba for tanning hides, as sun protection and as an insect repellant [73, 75]. While “contamination” of the human milk samples with *otjize* limits our ability to assess the maternal transfer of elements, our results reflect that Namibian infants are likely ingesting micronutrients through milk and suckling contact and very likely represent what these infants are typically consuming. Thus, we have chosen to not exclude the “contaminated” Namibian samples from analysis as, for this population, this composition is likely to be most relevant for considering infant outcomes. We do not yet understand if or how trace metals from *otjize* may be used by the infant or commensal microbes, but these results highlight the importance of evaluating mother’s milk within the context of cultural ecology.

Understanding the range of variation of essential metals in human milk will help to determine the most physiologically relevant concentrations to inform guidelines for supplementation and the production of infant formula. Levels of minerals in infant formula are often higher than in human milk because formula production must also consider differences in bioavailability and loss during production and storage [76]. However, the levels of minerals in formula can be much as ten times higher than in human milk [77]. Today, a variety of analytical techniques, such as ICP-MS, allow for sensitive, reliable, and cost-effective determination of multiple micronutrients from a small-volume sample [78]. There is now a growing body of literature to address the need to determine current concentrations of trace essential and toxic elements in populations around the world (*e*.*g*. [11, 12, 36, 48]). Further studies will be needed to add robust datasets from diverse populations to these new values.

## Conclusions

The concentrations of essential and potentially toxic elements in human milk are variable among populations. Due to small sample sizes, our study is limited in its ability to make definitive conclusions about the causes of these differences. However, our study is able to add information about the range of trace metal concentrations in diverse contemporary populations using modern, sensitive laboratory methods.

## Supporting information

S1 Dataset Human milk trace metal concentrations. (CSV)
